# The Future of Epidemic and Pandemic Vaccines to Serve Global Public Health Needs

**DOI:** 10.3390/vaccines11030690

**Published:** 2023-03-17

**Authors:** Andrew Farlow, Els Torreele, Glenda Gray, Kiat Ruxrungtham, Helen Rees, Sai Prasad, Carolina Gomez, Amadou Sall, Jorge Magalhães, Piero Olliaro, Petro Terblanche

**Affiliations:** 1 Nuffield Department of Medicine, University of Oxford, Broad St., Oxford OX1 3BD, UK; 2 Oxford Martin School, University of Oxford, Broad St., Oxford OX1 3BD, UK; 3 Independent Consultant and Institute for Innovation & Public Purpose (IIPP), University College London, London WC1E 6BT, UK; 4 Office of the President, South African Medical Research Council (SAMRC), Tygerberg 7050, South Africa; 5 Center of Excellence in Vaccine Research and Development (Chula Vaccine Research Center, Chula VRC), Bangkok 10330, Thailand; 6 School of Global Health (SGH), Faculty of Medicine, Chulalongkorn University, Bangkok 10330, Thailand; 7 Wits RHI, University of Witwatersrand, Johannesburg 2050, South Africa; 8 Bharat Biotech International Limited, Genome Valley, Shameerpet, Hyderabad 500 078, India; 9 Facultad de Derecho, Universidad Nacional de Colombia, Cra 45, Bogotá 111321, Colombia; 10 Virology Department, Institut Pasteur de Dakar, 36, Avenue Pasteur, Dakar 10200, Senegal; 11 Centre for Technological Innovation, Institute of Drugs Technology–Farmanguinhos, Oswaldo Cruz Foundation, Rio de Janeiro 21041-210, Brazil; 12 ISARIC Global Support Centre International Severe Acute Respiratory and Emerging Infection Consortium, Pandemic Sciences Institute, University of Oxford, Oxford OX1 3BD, UK; 13 Afrigen Biologics (Pty) Ltd., Cape Town 7441, South Africa

**Keywords:** vaccine access, platform technologies (esp. inactivated viruses; recombinant proteins; viral vectors; mRNA), technology global commons and global public goods, low- and middle-income country (LMIC) vaccine innovation and manufacture, epidemic and pandemic prevention, vaccine business model and value proposition, COVID-19 vaccine lessons, controlling transmission, variants and booster strategies, disease X and pathogen X, R&D blueprints, COVAX, regulatory challenges, mucosal immunity, pan-coronavirus vaccines, vaccine prototypes, modular vaccine production, vaccine intellectual property (IP) and know-how transfer, public investments and subsidies

## Abstract

This Review initiates a wide-ranging discussion over 2023 by selecting and exploring core themes to be investigated more deeply in papers submitted to the *Vaccines* Special Issue on the “Future of Epidemic and Pandemic Vaccines to Serve Global Public Health Needs”. To tackle the SARS-CoV-2 pandemic, an acceleration of vaccine development across different technology platforms resulted in the emergency use authorization of multiple vaccines in less than a year. Despite this record speed, many limitations surfaced including unequal access to products and technologies, regulatory hurdles, restrictions on the flow of intellectual property needed to develop and manufacture vaccines, clinical trials challenges, development of vaccines that did not curtail or prevent transmission, unsustainable strategies for dealing with variants, and the distorted allocation of funding to favour dominant companies in affluent countries. Key to future epidemic and pandemic responses will be sustainable, global-public-health-driven vaccine development and manufacturing based on equitable access to platform technologies, decentralised and localised innovation, and multiple developers and manufacturers, especially in low- and middle-income countries (LMICs). There is talk of flexible, modular pandemic preparedness, of technology access pools based on non-exclusive global licensing agreements in exchange for fair compensation, of WHO-supported vaccine technology transfer hubs and spokes, and of the creation of vaccine prototypes ready for phase I/II trials, etc. However, all these concepts face extraordinary challenges shaped by current commercial incentives, the unwillingness of pharmaceutical companies and governments to share intellectual property and know-how, the precariousness of building capacity based solely on COVID-19 vaccines, the focus on large-scale manufacturing capacity rather than small-scale rapid-response innovation to stop outbreaks when and where they occur, and the inability of many resource-limited countries to afford next-generation vaccines for their national vaccine programmes. Once the current high subsidies are gone and interest has waned, sustaining vaccine innovation and manufacturing capability in interpandemic periods will require equitable access to vaccine innovation and manufacturing capabilities in all regions of the world based on many vaccines, not just “pandemic vaccines”. Public and philanthropic investments will need to leverage enforceable commitments to share vaccines and critical technology so that countries everywhere can establish and scale up vaccine development and manufacturing capability. This will only happen if we question all prior assumptions and learn the lessons offered by the current pandemic. We invite submissions to the special issue, which we hope will help guide the world towards a global vaccine research, development, and manufacturing ecosystem that better balances and integrates scientific, clinical trial, regulatory, and commercial interests and puts global public health needs first.

## 1. Introduction

Developing many COVID-19 vaccines across multiple technology platforms in under a year and manufacturing and distributing billions of doses of them while leaving swathes of the world’s population without access in the face of a pandemic is both an incredible achievement [1,2,3] and a terrible indictment of our current health innovation model and of global collaboration and solidarity. An estimated 31% of the world’s population is yet to receive even one dose of a coronavirus vaccine and there are wide disparities [4]. In low-income countries (LICs), only 26% of the population have received a single COVID-19 vaccine dose compared to over 80% in high-income countries (HICs) [5,6]. Inequity has moved on to next-generation COVID-19 vaccines, tackling variants of concern (VOCs). HICs are rolling these vaccines out—despite limited evidence on their effectiveness—while LMICs utilise current batches of first-generation vaccines and will lag months to years behind HICs in access to vaccines that match circulating strains. While lauding the successes, we also need to recognise and tackle the limitations.

COVID-19 sits on a list of diseases including Ebola, Zika, Lassa fever, and a range of known others that the WHO considers high priority for research and development due to their potential to generate future epidemics and pandemics [7,8,9]. Recognizing that a pandemic could be caused by a pathogen currently unknown to cause human disease, the list also includes unknown disease X [10]. Pathogen X will most probably be a zoonosis, most likely an RNA virus, that emerges when the right mix of risk factors converge to allow pathogen X to move from animal populations to human populations to sustained human-to-human transmission [11,12]. The WHO has been working with hundreds of scientists on a series of R&D blueprints for high-risk pathogens [13,14]. Then there is influenza, which is probably the most likely next pandemic. There are mechanisms for flu surveillance, and flu vaccines are regularly updated to try to keep pace with circulating virus strains; however, such vaccines are not designed to meet LMIC needs [15]. LMICs have low rates of vaccination against seasonal influenza, and there is little progress in increasing coverage [16]. Shocking though it may sound, recent epidemics and pandemics might be milder versions of what the world will one day face, including—conceivably—from SARS-CoV-2 [17]. If we ever find ourselves up against a much more pathogenic virus and facing more scientific difficulties in developing a vaccine, our current approach will fail.

## 2. Tensions between Commercial and Economic Interests and Scientific and Public Health Interests

At the heart of current limitations sits the unresolved tension between, on the one hand, the commercial and economic drivers that underly our pharmaceutical innovation model and, on the other hand, scientific and public health interests. We want better vaccines against SARS-CoV-2 and other high-risk pathogens that are: safe; provide more potent and lasting immunity (including cell-based and potentially broad-spectrum immunity); block virus transmission; are easy to produce, transport, store, and administer, especially in resource-limited settings; and are accessible to all who need them. However, achieving these objectives will require us to resolve this tension.

The tension is exposed most obviously in the inequality of access to vaccines and of scientists from the global South to the means to research solutions for their own health needs. Early decisions, for example of US WARP speed [18,19], regarding which vaccines to promote over competing vaccines, focused largely on the registration needs of private companies and their proprietary technologies and quick regulatory approval in wealthy countries (which shaped clinical trial designs) over rapid, affordable, global access. Many HIC governments compounded this by trying to vaccine-protect their populations before all others, stockpiling spare doses and paying lip service to the WHO recommendation to vaccinate the most vulnerable everywhere first [20,21,22].

The founding documents of COVAX [23], the global mechanism set up in anticipation of this failure and hoping to avoid it, proposed reshaping the R&D process and centralizing procurement and allocation to address the inevitable initial scarcity. However, in its first year, COVAX itself struggled to move up the queue as richer countries bid for and hoarded doses (as many as ten doses per head of population) [24]. Vaccine scarcity was then artificially prolonged by the refusal to share technology. In 2022 and 2023, with the population of many LMICs having acquired (partial) protection by vaccination or infection and doses arriving too late to have much impact [25], the notion of investing heavily in mass vaccination with vaccines against the original COVID-19 strain made less and less sense. Vaccine equity is not about simply scaling up supply over time; it is about ensuring access to appropriate vaccines at the right time for optimal health impact [26]. This requires recipient, not donor, countries to shape product selection and procurement to serve their local requirements.

The tension distorts incentives away from epidemic prevention. From a public health perspective, a more efficient way to prevent pandemics is for vaccine research and development to tackle outbreaks when and where they arise—whether or not they can potentially become pandemics, and especially if they can. This means treating inequality in access to such technologies as a pandemic risk. Effectively addressing this threat requires the targeted utilization of small-scale interventions that are safe, effective, and adapted, and rarely the mass, high-profit, scaled-up production and deployment of health technologies. This means rapid-response R&D adapting existing vaccine technology platforms, rather than de novo approaches that take much longer. However, because there will always be risk of spillover and a broader spread, preparedness also requires stockpiling interventions that are never used or having the surge capacity to produce new interventions.

COVID-19, in creating a large market for PPR (Pandemic Preparedness and Response) interventions, is more the exception than the rule. Apart from HIV/AIDS, which (lacking a cure or preventive vaccine) has characteristics of a chronic disease that creates a large market opportunity, most epidemics are relatively short-lived and geographically limited, confined to underserved populations, and not representing an attractive market for pharmaceutical companies.

In response to SARS-CoV-2, heavy government subsidies—the direct financing of R&D and manufacturing capacity and the indirect risk-reduction value of advance contracts for billions of vaccine doses—enabled pharmaceutical companies to rapidly move vaccines to regulatory authorization and, for some companies, to sell them at premium prices significantly above the costs of production [27], generating huge profits (e.g., Moderna (Cambridge, MA, USA), Pfizer (New York City, USA)/BioNTech (Mainz, Germany)) even as others focused on (temporary) not-for-profit or low-profit approaches (e.g., Corbevax [28] or the Astrazeneca/University of Oxford vaccine [29]). The majority of the production of mRNA vaccines went to HICs able to pay tens of billions of dollars for supplies [30]. The subsidies, driven high by HIC bidding wars, were above the rewards needed to shield against risk. Instead of massive new funding instruments being used to help the industry from LMICs to partner with the industry from HICs in technology transfer and local production, they supported the already dominant firms, which were mostly not geared towards or interested in supplying LMICs.

The tension is evident in commercial strategies. Current COVID-19 vaccines were developed to target the spike protein of the wild-type SARS-CoV-2 virus identified at the start of the pandemic. Mutations changed the structure of this part of the virus, reducing vaccine efficacy [31,32]. Since September 2020, there have been five SARS-CoV-2 “variants of concern” (VOC): Alpha, Beta, Gamma, Delta, and Omicron, which quickly replaced previous variants and are now evolving into multiple, co-circulating sub-variants—a “swarm of variants… a variant soup” [33]—making it hard to predict coming waves of infection. The commercial strategy has been to shift to new-variant-targeting boosters, similar to the approach taken to seasonal flu; however, the validity of this strategy for disease control is questionable for COVID-19. Appetite for “booster” shots is moreover in decline in many parts of the world [34,35] and, currently, any available new-generation vaccines are going to HICs.

The tension is also evident in regulatory processes. For example, there has been significant discussion of vaccines that induce mucosal immunity [36,37,38,39]. By reducing viral replication on mucosal surfaces at the site of viral entry, such vaccines would contribute to a more rounded immune response, deploying all branches of the immune system (antibodies, cellular, and mucosa), and might be more variant proof [40]. SARS-CoV-2 is essentially a mucosal pathogen, and the threat of a future pandemic pathogen being mucosal and invasive is high. However, we have come to rely on vaccines that primarily induce humoral and cellular immunity in the blood instead of directly inducing mucosal immunity. In part, this occurred because the commercial race to licensure and the huge, time-limited subsidies embedded in contracts favoured trials that provided the quickest readout—the reduction of symptomatic, mainly mild-to-moderate infections—rather than trials that tested for stopping transmission. In the current and competitive commercial ecosystem, it is difficult to design contracts for vaccine purchase or R&D that incentivize companies to develop vaccines with specific characteristics, including “‘better” ones, if those take longer or are more challenging to develop. One problem is the lack of a clear regulatory pathway, as developers would need to design a clinical trial that could be compared with existing vaccines, and mucosal immunity is very different from what currently exists. A response might be to combine a mucosal vaccine with a systemic vaccine delivered through the intramuscular injection route. However, the whole vaccine development ecosystem, including regulatory pathways, is not designed or equipped to develop and assess products in combination, and companies have no incentive to do so in a commercial context that favours competition over collaboration. No mucosal vaccines have made it to the licensure stage, although two, developed by CanSino Biologics and Bharat Biotech, have received emergency use listing in China and India, respectively [41].

While regulators have offered fast-track approaches for companies developing follow-on vaccines—new Pfizer and Moderna bivalent vaccines are being approved by immune-bridging studies—second-generation developers, including those in LMICs, face regulatory hurdles. Even if any developer could, in principle, make new vaccines that satisfy regulators, newcomers may still be required to produce clinical evidence of safety and efficacy. Moreover, there is no regulatory pathway for “generic” vaccines: the only option is to produce an existing vaccine under license from the originator.

To accelerate product development and comparability studies, we need also better correlates of protection for SARS-CoV-2 that are able to assess protection against severe disease, transmission, or long COVID [42,43]. Efficacy models will be required for novel technologies and constructs, but an understanding of the immune response that is predictive of protection could facilitate more rapid licensure of new vaccines on existing platforms and constructs [44]. Because one size does not fit all, we must design regulatory guidelines and strengthen capacity to specifically address the regulatory challenges faced by developers in LMICs, including ensuring that benefit–risk weighing is fit-for-purpose to the local health system context. For instance, a product that needs to be stored and transported at ultra-low temperatures, has a short shelf life, or requires intensive post-marketing surveillance might not be suited for use in low-resource settings.

## 3. Designing Future Epidemic and Pandemic Vaccines

To progress beyond variant chasing, efforts are underway to design vaccines that recognize several coronaviruses, including the seven known human coronaviruses—the four that cause seasonal, mild upper respiratory tract infections and the three that cause lower respiratory tract infections that can progress to acute lung injury and death (Middle East Respiratory Syndrome Coronavirus, MERS-CoV; Severe Acute Respiratory Syndrome, SARS-CoV-1; and SARS-CoV-2, which causes COVID-19). The next step—the “holy grail” as some have called it [45]—is a broadly protective pan-coronavirus vaccine offering protection against any variants or sub-variants of SARS-CoV-2 that might ever emerge and any future emerging coronaviruses [46,47,48,49]. This would exploit a part of the virus that does not mutate over time—a “conserved site”—such as a part of the virus that binds to a specific protein on the targeted host cell [50,51]. The hope is that this site is present on more than one virus and will become the “Achilles’ heel” of all coronaviruses. However, numerous hurdles slow clinical progress [52].

SARS-CoV-2 is just one of many threats. To deal with threats from multiple pathogens, the Coalition for Epidemic Preparedness Innovations (CEPI) and the National Institutes of Health (NIH) have worked on identifying vaccine prototypes for each group of pathogens, with the aim of having a repertoire ready for phase I/II trials if ever needed [53]. A corollary need is to integrate pathogen genomics into existing public health surveillance systems [54]. However, without changing the underlying business model, any notion of a 100-day mission [55] risks being mostly about having vaccines available more quickly in HICs. Perversely, this would undermine HIC health security when rapid global access for all is needed. Given the number of potential pathogens and vaccine platforms, it will still be necessary to select, by an agreed methodology, which pathogens to prioritize, perhaps by region, and how many platforms to work on per pathogen. It would remain challenging to mount an equitable global roll-out in the face of an especially transmissible and virulent pathogen.

## 4. Sharing Vaccine Platform Technology and Know-How to Strengthen Global Vaccine Capacities

Four platform technologies are the basis of most current vaccines: inactivated viruses; recombinant proteins; viral vectors; mRNA [56]. The tried-and-tested approach for each vaccine developer is to adapt its proprietary technology to the latest pathogen or its variant, for instance by updating the vaccine based on prevalent viral strains (such as for seasonal influenza), or by adding more antigens to cover more strains (such as for the pneumococcal vaccine) or a new genetic variant (such as Moderna’s updated COVID-19 vaccine), or by switching to a different target/virus using the same technology platform (such as the Johnson & Johnson and University of Oxford vaccines originally developed for Ebola [57] and MERS [58], respectively, which both switched to SARS-CoV2, and IAVI’s Ebola Sudan virus vaccine, based on Merck’s Ebola Zaire vaccine, which originated from Canadian public research [59]).

The key to protecting future global public health will be the management of these technology platforms as shared global commons or global public goods that are non-proprietary and available for any developer of epidemic and pandemic vaccines [60]. Instead of companies competing to achieve the biggest share of the market based on a monopoly control of these platforms, slow or no technology transfer, and delayed access in many parts of the world, there would be agile, decentralised, and localised innovation and multiple manufacturers in many places able to plug in new variants or pathogens. Low-cost and more flexible technology platforms—such as mRNA (which does not require bioreactors, which carry a high risk of failure), and protein-subunits, as well as particle vaccines that mimic components of viruses and bacteria to induce protective immunity—are well suited to lower-resource settings. There may be an initial phase during which costs are higher, and mechanisms will need to be found to sustain LMICs through this phase. We learned from COVID-19 that heterologous prime-boosting enhances the immune response [61,62]; therefore, we must find ways to promote the use of different platforms in various combinations.

Around the world, several initiatives aim to boost capacity for vaccine manufacture and, increasingly, for vaccine research and development in LMICs [63]. LMIC governments, with the support of HIC donors, philanthropies, and development banks, have supported vaccine production at varying levels, such as by providing land, tax incentives, infrastructure, and finance to public–private partnerships. Of note, in South Africa, Cuba, Brazil, India, and elsewhere, governments have been investing in their National Regulatory Authorities (NRAs) with more training, the qualification of GMP inspectors, quality control of laboratories, and vaccine and technical expertise to perform lot release and to support collaboration with competent authorities of other countries and the WHO. In Africa, there is very active engagement of the Africa CDC [64], the African Development Bank (which recently launched the African Pharmaceutical Technology Foundation [65]), and African vaccine manufacturers themselves [66,67]. The new African Medicines Agency aims at regulatory harmonization. Twenty-five percent of the global use of vaccines occurs in Africa, yet African companies produce less than one percent of vaccines used across the continent [68,69]. The African Union has set the goal of producing sixty percent of vaccines delivered across Africa in Africa by 2040. Despite there being several vaccine companies in Africa, they contributed little during the pandemic, even as companies in other LICs were able to innovate, develop, and manufacture vaccines at scale. We need to better understand why this was the case and how to overcome barriers and build a sustainable African vaccine research, development, and manufacturing ecosystem.

To counter national pressures to vaccinate local populations before supplying doses beyond domestic borders, we might explore “small country solutions”. These might include select, distributed networks of small nations to scale the manufacture of emergency vaccines quickly, supported at least by a voluntary pooled licensing scheme similar to the UN-supported Medicines Patent Pool (MPP) [70] or the COVID-19 Access to Technology Pool (C-TAP), [71] but ideally by comprehensive technology and know-how sharing via initiatives such as the mRNA vaccine technology transfer hub (see below) [72]. Equipping local research and manufacturing teams with the technology and skills should allow for rapid-response R&D based on adapting shared platform technologies. Flexible, modular pandemic vaccine production systems (such that once a microbial threat is identified and sequenced, sites in affected regions can develop their own versions and manufacture their own supplies of vaccine with relative swiftness) might be part of regional containment strategies [60]. Technology transfer pathways with LMICs might be seeded by sharing vaccine-specific knowledge and know-how through milestone-driven partnerships with vaccine centres of excellence (such as the Sienna-based GSK global vaccines facility), and early transfer at proof-of-concept and at later stages of clinical development. This might involve pilot facilities for early clinical development and scaling up the market size over time.

## 5. Overcoming Hurdles in the Way of LMIC Vaccine Developers and Manufacturers

There are many examples worth exploring, one of which is the WHO-supported mRNA vaccine technology transfer hub launched in South Africa in June 2021 (Afrigen Biologics & Vaccines, South African Medical Research Council, Biovac). When companies and governments did not share their knowledge and technology [73], Afrigen set out to reverse-engineer and adapt Moderna’s COVID-19 mRNA vaccine using information already in the public domain with support from a network of scientists in South Africa and elsewhere. The hub is sharing the technology with manufacturers in 15 countries, enabling them to not only produce the initial COVID-19 vaccine but also to adopt mRNA technology to innovate according to their own health needs. Nevertheless, though Moderna has pledged not to enforce its patents in a limited number of LMICs during the pandemic, it is risky to build capacity on the basis of a need that might no longer exist, in a market that might one day be deemed “non-pandemic”, and in technology over which the freedom to operate might be blurred by patent litigation and/or voluntary commitments that are not enforceable.

Meanwhile, Moderna, Pfizer, and BioNTech are setting up local production facilities in various LMICs, typically only fill-and-finish facilities that use ingredients delivered from facilities in Europe, treating local companies as the last part of the supply chain [74], litigating each other over the ownership of IP [75] and pressuring wealthy countries against granting waivers on certain IP rights as proposed to the World Trade Organization by South Africa and India.

The impossibility of building a sustainable LMIC vaccine industry that relies on deals with HIC companies based on the manufacture of only COVID-19 vaccines under license is highlighted by the recent halting of production at Aspen Pharmacare, Africa’s largest COVID-19 vaccine manufacturing plant, which had signed a contract to fill-and-finish approximately 180 million doses of the J&J single-shot COVID-19 vaccine. At first, the EU bought up most doses to cover a shortfall caused by manufacturing problems at Emergent BioSolutions in the US. Then, because the J&J vaccine fell short compared to other vaccines, demand for it collapsed, leaving J&J with millions of spare doses stockpiled. In August 2022, the Serum Institute of India signed a 10-year deal to utilize Aspen’s near-idle COVID-19 vaccine production lines to manufacture four routine paediatric vaccines used in Africa based on bulk ingredients supplied by the Serum Institute [76]. Meanwhile, the Serum Institute has 200 million stockpiled doses of Covishield, the Oxford/AstraZeneca vaccine, and has stopped manufacturing any more. We have moved to the next level of inequity wherein any oversupply of older-variant vaccines is distributed to the have-nots or destroyed, and rich countries sign contracts to hog the latest-variant vaccines [77].

Without more distributed and equitable vaccine development and manufacturing capacity, many LMICs, and especially LICs, will remain dependent on HIC producers that prioritize HIC markets, and at the back of the queue when global pandemics strike. Vaccine research, development, and manufacturing capacity in LMICs will only be sustained, researchers and technicians will only be trained for product-specific manufacturing and vaccine quality control, facilities will only be occupied, and all technology platforms will only be kept “warm” if vaccine R&D capacity and cutting-edge local science are sustained to develop and make many other vaccines in interpandemic periods and by making equitable access to effective vaccines an explicit economic, industrial, and sustainable development goal (SDG).

There is also a need for the concrete integration of pharmaceutical manufacturing development into economic development planning. Ethiopia has published a pharmaceutical manufacturing development plan [78]. The African Union has issued the Pharmaceutical Manufacturing Plan for Africa (PMPA) [79] and an ambitious 50-year plan for the continent [80]. Other LMICs might usefully explore the trajectory of Brazil, whose government implemented a strategic health–industrial development plan through which it supported cooperation, by agreements between public and private institutions, for the development, deployment, transfer, and absorption of technology relating to products of strategic importance, beginning with Brazil’s own needs and working outwards to the needs of the world [81,82]. Since vaccine development and manufacturing projects might need several years to be established, stable political and economic environments in countries where facilities are or will be located is also essential.

## 6. Towards a Global Public Health “Value Proposition”

At the heart of our problem lies a fundamental dichotomy. Effectively dealing with outbreaks, whatever their scale, geographical location, spread, or the purchasing power of those affected, will serve global public health needs, ensure equity, and be the most effective first line of defence against pandemics by “nipping them in the bud”. However, this pursuit is not aligned with commercial interests. Our current pharmaceutical business model needs a large expected market size to generate commercial incentives and will never respond in ways that allow us to tackle smaller outbreaks. The only way forward is to change the narrative from “business model” to “value proposition” and from “market-driven” to “public-health driven”.

Once the current pandemic funding is gone, a different investment model will be required to ensure we can develop, produce, and make equitably available the vaccines we need for global public health. What structural, ownership, regulatory, or other changes will this entail? How will different investors, including the public, be rewarded a fair share of the total “returns”? Most of the massive COVID-19 public investment—paid for through taxes and philanthropy, not to mention the efforts of numerous global health researchers and people and communities agreeing to be part of clinical trials—has gone to boost the profits of a handful of companies in HICs, with Pfizer/BioNTech and Moderna topping the list with an estimated USD 34 billion profit in 2021 alone [83]. Commercial returns surely have a place. The key is to distribute the risks, costs, rewards, and profits more efficiently between different stakeholders in the value chain so that all returns are not given only to one stakeholder; to separate out those parts that are clearly global commons or global public goods [84,85], such as platform technologies, from those parts that are not; for commercial returns to be based on equitable access to those technologies; and for the overriding goal to be the maximum impact on global public health [86].

Examples we might explore include: a specific fund to support LMIC developers and manufacturers, especially through the early phases of capacity strengthening; strategies to realize regional capabilities, including locally manufacturing active ingredients and pooling local demand; investment in manufacturing technologies that are highly efficient at small and medium scales and can be modulated to produce more as needed and switched to another product depending on need; mechanisms and funding to support those especially well adapted to respond to small “local” outbreaks (perhaps as an “outbreak response” function on top of a “routine vaccine response” function); changes in the practices of procurement agencies (UNICEF, GAVI, etc.) and LMIC procurement policies to support local development and manufacturing efforts, for example, by carving out a set percentage of their procurement for LMIC companies as a means of reassuring investors in regional development and manufacturing hubs; ex-ante capacity and supply agreements to stop the political scramble to be the first to get available supply; and replacing pandemic finance mechanisms based on pandemics exploding before a pay-out is triggered (notably, the insurance window of the World Bank’s now-defunct Pandemic Emergency Financing Facility, PEF [87]) with mechanisms focused on epidemic prevention that support multipolar, regional innovation hubs with the capacity, know-how, and freedom to operate to adapt existing technology platforms to deliver locally needed health technologies.

Governments, research funders, and development banks will need to leverage their huge investments [88] and risk-bearing services to extract binding, enforceable commitments to equitable access and the sharing of critical technology so that many more countries can engage in vaccine development and manufacturing to fight epidemics and pandemics. The Independent Panel for Pandemic Preparedness and Response has talked of “pre-negotiated systems for a global health commons approach to mobilize pandemic tools” [89], end-to-end R&D platforms, predictable financing, adequate industrial policies, inclusive governance, and regional platforms.

A proposal has been put forward regarding six interlinked building blocks that create an ecosystem rooted in equity at every stage from research through to delivery [60]. Such a system, with governance and financing negotiated in advance, would promote open sharing and regional resilience, and would be rooted in a global commons approach. There is also talk of aligning biomedical R&D with global public interests [90,91], and of a pandemic treaty [92,93,94] that obliges countries to support, for the global public good, the free flow of goods, services, and knowledge for tackling epidemics and pandemics.

We will not turn such aspirations into practical realities by tinkering in the margins. Instead of time and again trying to fix market failures on a tilting playing field, we need to change the narrative from “business model” to “value proposition”. If the goal is to create new and adapted vaccines that are equitably accessible for all around the world, we must reorganize the health innovation and production ecosystem and the interactions between public and private stakeholders to achieve that goal through appropriate economic, industrial, health, procurement, fiscal, and other policies.

## 7. Vaccine Priorities Served by a Global Public Health Value Proposition

A good starting point for considering what “desirable or appropriate epidemic and pandemic vaccines” would look like is to ask what the goal of vaccination programs should be from a global public health perspective, which will vary according to the disease and the nature of an epidemic and will extend beyond the WHO’s current Target Product Profile for COVID-19 vaccines, which prioritizes short-term needs and assumes a constrained technology environment [95]. The list includes at least the following:

- Vaccines that break transmission. Global public health is about the global impact of vaccines, yet the COVID-19 vaccine announcement-by-press-release [96] largely focused on vaccine efficacy for the individual person. This caused policy disconnects, with vaccines that were good at preventing disease progression in the individual being used to cut population-level transmission without knowing if they could. The speed of the emergence of variants, taking advantage of the still-wide circulation of SARS-CoV-2, even in highly vaccinated populations, indicates that this is not working and leaves the world vulnerable to more virulent variants.
- A pathway towards improved vaccines that do not just do more of the same as the first-generation. Target product profiles everywhere need to be dynamic and evolve as epidemics evolve; what we expect and need from a vaccine today may be very different from what we needed at the start of the pandemic because many people now have some immunity through vaccination, natural infection, or both.
- A long-term, sustainable strategy for dealing with mutations, not only of SARS-CoV-2 but of all future potentially highly mutable pathogens. It should have been no surprise that SARS-CoV-2 mutates frequently. Other coronaviruses, such as those for the common cold, do so all the time. However much surveillance improves and however quick the vaccine development process becomes, the current approach can only ever benefit a few.
- Strategies that work in different health contexts, including resource-limited contexts but also contexts with different epidemiolocal parameters. Many current strategies “work” imperfectly in HICs and, even then, only because HICs are prepared to keep throwing money at the problem. The apparent success in HICs creates complacency that harms LMICs who cannot afford such luxury or need better-adapted tools.

## 8. A New Global Approach to Vaccine Research, Development, and Manufacture Is Needed

The apparent success of vaccines against COVID-19 has lulled us into a false sense of security. Many new pandemic initiatives, while accepting the need for change in areas such as surveillance, are much less vocal for the need for change in areas such as vaccine research, development, and manufacture, and avoid tackling issues of ownership and control, access to technologies, and open science.

We need to recognize the highly convoluted, multiple-horizon, multiple-investment decision problem we face. We need to break the messy process into its components and identify the different actors and how their actions and incentives might be changed. Change is more likely to come from a political movement for change that is based on all stakeholders coming together—LMIC developers, researchers, beneficiaries of improved global public health, governments, public donors, and funders who come to understand how a different approach will improve global public health security.

We have experienced three coronavirus outbreaks in the last 20 years: the 2002–2004 SARS-CoV-1 outbreak, MERS-CoV in 2012, and now SARS-CoV-2. The WHO counts nearly 30 Ebola virus disease outbreaks since 1976 that involved major or massive cases [97]. In 2022 and 2023, despite all the supposed COVID-19 lessons, the same mistakes and deficiencies observed with Ebola virus disease (EVD) and COVID-19 [98] are being repeated with mpox [99,100,101].

The mpox treatment (tecovirimat) and vaccine (MVA-BN) were developed in recent years, motivated and financed through the US health security programme to protect against smallpox. Their development was incentivized by several “market push-and-pull” mechanisms including the significant public financing of R&D, regulatory incentives, and generous purchase commitments to support stockpiling. They are registered in several HICs; however, despite recurrent outbreaks, they are not available in mpox-endemic African countries (except as an expanded-use programme [102] and a trial of tecovirimat [103]). Only when mpox spilled over to HICs and was declared a PHEIC (public health emergency of international concern) did the vaccine and treatments suddenly become available (though still not in African countries). Not tackling viruses like mpox where they occur is a failure in health equity and opens the door to major spillovers to the rest of the world.

It is inevitable, based on recent trends, that there will be future virus outbreaks, including outbreaks of coronaviruses [104]. Pandemic influenza is also a high probability. The next time could be much worse than this time. All the recent wealth of vaccine research and development and capacity building could help prevent epidemics and pandemics. This will only happen if we question all prior assumptions, stop paying lip-service in high-level meetings and summits to global cooperation and equity, and start focusing on translating technological progress more rapidly into effective public health impact for all, including equitable access to the means of vaccine research, development, and manufacturing. This Review is intended to initiate a debate. Over the coming months, the special issue of *Vaccines* will gather together the expertise of a global community of researchers and practitioners to turn lessons learned from the current pandemic into a much-improved response to future epidemics and pandemics.

## Data Availability

No new data were created or analyzed in this study. Data sharing is not applicable to this article.
